# Non-invasive assessment of positive affective state using infra-red thermography in rats

**DOI:** 10.1017/awf.2023.87

**Published:** 2023-09-29

**Authors:** Chanakarn Wongsaengchan, Dominic J McCafferty, Katie Lennox, Ruedi G Nager, Dorothy EF McKeegan

**Affiliations:** 1 School of Biodiversity, One Health & Veterinary Medicine, University of Glasgow, Glasgow G12 8QQ, UK; 2 School of Psychology & Neuroscience, University of St Andrews, St Andrews, KY16 9JP, UK

**Keywords:** animal welfare, positive welfare, rats, reward magnitude, sex difference, thermal imaging

## Abstract

With recent increased focus on positive welfare in animal welfare science, there is demand for objective positive welfare indicators. It is unclear whether changes in body surface temperature can be used to non-invasively identify and quantify positive states in mammals. We recorded continuous measurements of tail surface temperature using infra-red thermography (IRT) and concurrent behavioural observations in male and female Wistar rats (*Rattus norvegicus*). If tail surface temperature can differentiate between positive and negative experiences, we expect a qualitatively different response compared to negative experiences. Three groups of rats were presented with increasing magnitudes of food rewards (neutral/none, one and three rewards). The rats were placed in an arena to which they were habituated and filmed for 30 s before and 30 min after exposure to different rewards. Tail temperature initially decreased from the pre-reward baseline and subsequently returned towards baseline temperature. The overall pattern of the change was the same as for rats subjected to negative stimuli in previous studies. Nevertheless, dynamic changes in tail temperature, specifically the rate of recovery and the behavioural response (exploration), differed between neutral and rewarded rats but failed to distinguish reward magnitude. Sex differences were found in both thermal and behavioural responses, unrelated to reward magnitudes. Female rats exhibited a greater initial response with a slower recovery than male rats, emphasising the value of using of both sexes in animal welfare research. This study improves our understanding of the effects of positive emotions induced by food reward on peripheral body temperature and behaviour.

## Introduction

In animal welfare science, the focus is shifting from assessing and avoiding or reducing negative experiences and suffering in animals (Broom & Johnson 1993; Farm Animal Welfare Council [FAWC] 2009; Hawkins *et al.* 2011; Animals in Science Committee 2017) to also identifying and promoting positive welfare states (Boissy *et al.* 2007; Yeates & Main 2008; Mellor 2016; Lawrence *et al.* 2019). Furthermore, to adequately address the complex nature of animal welfare, recent animal welfare concepts have incorporated the individual adaptability of animals and taken the dynamic nature of animal welfare over time into account (McMillan 2019; Arndt *et al.* 2022). Therefore, there is an increasing requirement for objective and reliable welfare indicators that are sensitive to the dynamics of positive affective states of animals.

Identifying an animal’s affective state is one of the biggest challenges in animal welfare science, the well-established approaches of identifying affective state using concomitant behavioural and physiological measures have been the basis of significant progress in animal welfare research (Möstl & Palme 2002; Olivier *et al.* 2003, Buynitsky & Mostofsky 2009; Mendl *et al.* 2009; Hubrecht & Kirkwood 2010; Campos *et al.* 2013). According to the dimensional perspective of affective states, the most commonly assumed dimensions are valence (negative or positive) and arousal (high or low) (Mauss & Robinson 2009). The valence dimension contrasts states of pleasant (e.g. happy) with states of unpleasant (e.g. sad), and the arousal dimension contrasts states of low arousal (e.g. boredom) with states of high arousal (e.g. startle). Available physiological and behavioural measures of affective state differ in their sensitivity to arousal and valence, with behavioural responses being currently the main route to identify valence (Mellor 2015). Most available physiological measures lack valence and require invasive procedures (e.g. blood/tissue sampling, insertion of probe or surgical implantation of a data logger) that can impede the animal’s ability to freely express their behaviour and impact on affective state in itself (Burgdorf & Panksepp 2006; Boissy *et al.* 2007; Mendl *et al.* 2009; Wöhr & Schwarting 2009; Zupan *et al.* 2016; Duarte & Pinto-Gouveia 2017; Alexander *et al.* 2021). Infra-red thermography (IRT) has recently emerged as a promising non-invasive physiological measure that may provide information on an animal’s affective state (Travain & Valsecchi 2021).

Changes in body temperature (a rise in core temperature and concurrent drop in body surface temperatures) reflect the stress-induced hyperthermia (SIH) phenomenon and is triggered via the sympathetic activation of the autonomic nervous system (ANS) (Diorio *et al.* 1993; Oka *et al.* 2001; Olivier *et al.* 2003; Bouwknecht *et al.* 2007; Hänsel & von Känel 2008). Consequently, changes in body surface temperature measured with infra-red thermography have been used to non-invasively detect negative affective states in many endothermic species (for a review, see Travain & Valsecchi 2021). In systematic validation work, IRT has also been shown to be useful for quantifying arousal intensity of restraint stressors in hens (*Gallus gallus domesticus*) (Herborn *et al.* 2015) and in laboratory Wistar rats (*Rattus norvegicus*) (Wongsaengchan *et al.* 2023). However, less is known about ANS responses to positive affective states, although some responses have been reported to be valence-specific (Kreibig 2010; Shiota *et al.* 2011; Wilhelm *et al.* 2017; Ishii & Shinya 2021), including skin temperature responses (Kreibig 2010; Ioannou *et al.* 2014).

Few studies have used IRT to evaluate body surface temperature responses to positive stimuli. Studies in humans and non-human primates reported different directions (cooling/warming) of surface temperature of nose and fingers in response to negative and positive stimuli (Merla & Romani 2007; Kreibig 2010; Hahn *et al.* 2012). In monkeys and apes, upper lip temperature rose after negative stimuli while nose temperature decreased, and eye temperature increased after positive stimuli (Chotard *et al.* 2018). This suggests that the direction of temperature change could differ according to the valence of the response, and that discrete emotions may induce different autonomic patterns as supported by some authors (Larsen *et al.* 2008; Mauss & Robinson 2009). In domestic dogs (*Canis familiaris*), a positive event (receiving food treats) led to an increase in eye temperature (Travain *et al.* 2016). However, a pilot study found that a dog’s eye temperature also increased during a negative event (standardised veterinary examination) (Travain *et al.* 2015). Similarly, in chickens, a decrease in peripheral temperature was noted both when anticipating a positive event (Moe *et al.* 2012) and during a stressful situation (Edgar *et al.* 2013; Moe *et al.* 2017). Nasal temperatures decreased in response to positive experiences in cows and non-human primates (Proctor & Carder 2015; Chotard *et al.* 2018), again the same as reported in negative experiences (Nakayama *et al.* 2005; Ioannou *et al.* 2015; Kano *et al.* 2016; Heintz *et al.* 2019). The extent to which changes in surface temperature may reflect changes in arousal (i.e. high or low) and/or valence (i.e. positive/pleasant or negative/unpleasant) is still not clear.

Rats have been used extensively in studies of emotions, where the focus has been on applying the results to humans, however this process has yielded a great amount of information on rat physiology, behaviour and welfare (Makowska & Weary 2013). Rat tails are well-vascularised with arterio-venous anastomoses and lack fur, thus providing a suitable region of interest (ROI) for IRT (Gemmell & Hales 1977). Previous work in mice (*Mus musculus*) and rats including ours (Wongsaengchan *et al.* 2023) indicates that tail temperature should decrease in response to negative stimuli (Vianna & Carrive 2005; Marks *et al.* 2009; Reis *et al.* 2011; Fassini *et al.* 2014, 2017; Lecorps *et al.* 2016; Gjendal *et al.* 2018; Miyazono *et al.* 2018) and magnitude of stress is reflected in peripheral control of circulation in the tail (Wongsaengchan *et al.* 2023). There is no indication yet whether the experience of positive emotions would have a different effect on tail temperatures compared to negative emotions (valence), and whether different magnitudes of positive experiences (arousal) are reflected in differences in tail temperatures as is the case for negative experiences. While spontaneous behavioural tests, such as the open field test and elevated plus maze test, are commonly used in the study of animal negative emotion to standardise observational and stimulus techniques (Steimer 2011), the equivalent tests for positive emotions have not yet been validated. Play behaviour (Held & Špinka 2011), anticipation of a reward (Spruijt *et al.* 2001), 50-kHz calls (especially when being tickled or during social play) (Boissy *et al.* 2007; Hinchcliffe *et al.* 2020), facial expression (Finlayson *et al.* 2016) and optimistic cognitive bias (Mendl *et al.* 2009), have all been linked to positive emotions in rats. In specific contexts where other indicators of positive emotions (e.g. play and anticipation) are not available, such as during open-field IRT filming, the simplest spontaneous approach and avoidance behaviour may be used to gauge the general valence (negative/positive) of a stimulus: while freezing, darting, attacking behaviours may reflect negative emotions, exploratory and consumptive behaviours may indicate specific, object-directed positive emotions (Paul *et al.* 2005). The use of spontaneous approach and avoidance behaviour was used in our previous study to validate IRT responses, cross-validated with hormonal and behavioural responses (Wongsaengchan *et al.* 2023).

In this study we used food reward to induce positive affective state and we aimed to assess whether tail surface temperature measured non-invasively with IRT can identify positive affective state (valence) in male and female rats in response to a food reward and whether IRT can also quantify increasing levels of reward magnitude (arousal). We used Honey Cheerios (Nestlé®, UK), a highly palatable sweet cereal, conventionally used as food reward in rats (Makowska 2016; Makowska & Weary 2016; Brydges & Hall 2017; Nip *et al.* 2019). We collected concurrent thermal and spontaneous behavioural responses for cross-validation. Since sex differences have been inconsistently reported in the responses of rats to food reward (Marshall *et al.* 2017; Sinclair *et al.* 2017; Chowdhury *et al.* 2019), both sexes of rats were studied. We hypothesised that the tail surface temperature response to a food reward would differ between non-rewarded and rewarded rats and between rats exposed to different reward magnitudes as reflected by behavioural indicators of positive emotions in rats. Our objective was to provide evidence whether the body surface temperature can provide information on the valence and arousal of positive experiences in rats.

## Materials and methods

### Study animals and husbandry

All experimental procedures and data acquisition were carried out under UK Home Office authorisation (Project licence: PIFD5B3DB, Personal licence: I3D10B21C). The design and report of the study followed the ARRIVE (Animal Research: Reporting of *In Vivo* Experiments) guidelines 2.0 (Percie du Sert *et al*. 2020) and PREPARE (Planning Research and Experimental Procedures on Animals: Recommendations for Excellence) guideline (Smith *et al.* 2018) for reporting research in animals. Eighteen male and 18 female five week old dam-reared outbred albino Wistar rats (101–125 g on arrival, total n = 36) were acquired from Charles River (UK). By the time of testing, rats were 8 weeks old and had a mean (± SD) body mass of 257.97 (± 17.72) g (males) and 182.67 (± 10.21) g (females). Rats were housed in groups of three individuals of the same sex in a 48 × 37.5 × 21 cm (length × width × height) polycarbonate cage (Tecniplast, London, UK) and were maintained in a 12:12h light: dark cycle with lights on at 0700h. The mean (± SD) temperature and relative humidity of the room were 22.04 (± 1.95)°C and 55 (± 10)%, respectively. Animals had free access to *ad libitum* water and food (Maintenance and breeder pellets, CRM Special Diet Services, Witham, UK). In each cage, there was approximately 7-cm deep corn cob and sizzle nest bedding for burrowing, two cardboard tunnels and a 21.5 × 21.5 × 12.5 cm Sputnik rat house enrichment device (SAVIC nv®, Belgium). Rats were handled as part of normal husbandry with non-aversive handling tunnels (NC3Rs 2013) and were individually marked with a non-toxic animal marker (Stoelting Co, USA) at the end of the first habituation trial. All rats were inspected daily and found healthy. After the trials, the minority of rats were humanely euthanased and the majority retained and re-used under another Project Licence after veterinary certification of fitness.

### Habituation phase

**Figure 1.**
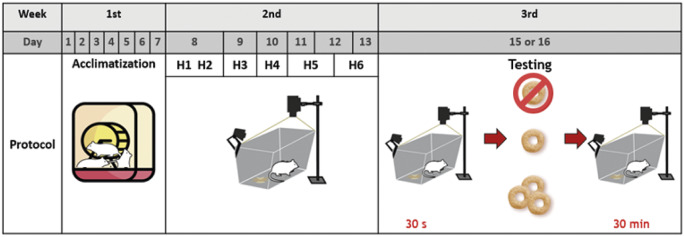
The three phases of the experimental protocol: acclimatisation; habituation; and testing. Rats were left undisturbed for the first week after arrival to acclimatise to the home cage. In the second week, six habituation trials of increasing duration of exposure to the test arena (5, 5, 10, 20, 30, 30-min duration represented as H1–6, respectively) were completed within six consecutive days; trials H1 and H2 were completed in one day and trials H5 and H6 were completed over three days. In the third week, each rat was tested by being put into the test arena and filmed with infra-red and video cameras for 30 s to record the baseline temperature and behaviour. Each rat was then exposed to one of three treatments (0, 1 or 3 Cheerios) and further filmed for 30 min.

The experimental protocol consisted of three phases: acclimatisation; habituation; and testing (Figure 1). Upon arrival rats were put into home cages in groups of three of the same sex and left undisturbed for seven days to acclimatise to the housing unit. Following the acclimatisation, rats were habituated individually to the testing conditions. Since we recorded the surface temperature of rats using IRT and infra-red radiation cannot pass through the polycarbonate base of the home cages and the wire-mesh cover obstructs a clear view of the rat, we recorded individual thermal response to different treatments in a separate test arena. During the habituation and the testing periods, rats were transferred individually from their home cage to the test arena which was located in a separate room. The habituation period was designed to overcome, as far as possible, the stress response to temporary social isolation, the transportation, the novel testing arena and the novel food reward. Transfers were carried out using a transport cage (a white opaque polypropylene rat cage sized 56 × 38 × 17 cm; North Kent Plastic Cages, UK) covered with a raised wire lid, supplemented with a handful of the rats’ own cage bedding material. Rats were tunnel-handled between home cage, transport cage and testing arena. The familiar odour of the bedding material was intended to minimise novelty and maximise habituation (Wallace *et al.* 2002; Burn 2008). Rats were put into the test arena along with the bedding material from their home cage. The test arena was a grey, plastic, open-topped box (40 × 30 × 32.5 cm; Key Industrial Equipment™, Napa, CA, USA). To allow recording of infra-red radiation without any obstruction of the view, there was no lid on the testing arena. In cases where a rat jumped up to the top edge of the testing arena, it was immediately tunnel-handled back into the arena. The habituation trials mimicked the testing period except that the animal was not given a food reward and the duration of exposure to the test arena was gradually increased (5, 5, 10, 20, 30, 30-min duration represented as H1–6, respectively) across the six habituation trials and completed within six consecutive days for each rat (Figure 1). Each habituation trial included putting the experimenter’s hand into the arena once every trial to habituate rats to the experimenter’s hand which would be the mean to introduce food reward into the arena in the testing period. Two transport cages and test arenas were used alternately and were cleaned between rats and trials using tap water and alcohol disinfectant wipes (Medipal®, Pal International Ltd, UK). The day before the beginning of the habituation period and at the end of the habituation period, rats were given the food reward in their home cage in order to minimise the novelty fear of the food reward in the testing period. The habituation protocol was assessed as a separate study and significant reduction of thermal and behavioural responses to being exposed alone in the testing arena over the six trials of increasing duration were observed (Wongsaengchan 2022). All habituation was undertaken during the light phase from 0830–1730h, i.e. within 1.5 to 10.5 h after the onset of the light phase.

### Testing phase

Testing phase began one day after the habituation period was completed (Figure 1) and was conducted 2.5–7.5 h after the onset of the light phase. Systematic randomisation was used so that each of the three rats within each home cage were exposed to a different treatment. One- or three-jointed Cheerios (Honey Cheerios cereal, Nestlé®, UK) were used as two different reward magnitudes according to a previous report that rats preferred a reward which is higher in density and surface area (Wadhera *et al.* 2018). Rats that were offered no reward served as a neutral (control) group. The first 30 s in the test arena were used to obtain individual baseline measure for body surface temperature and behaviour. Then one of the three treatments either neutral (zero food reward), one piece of Cheerio or three-jointed pieces of Cheerios were placed to the bottom of the arena by hand. For the neutral group, the experimenter put an empty hand into the arena to control for the effect of rats’ exposure to a hand to be the same throughout the three groups. After the treatment was applied, the rat was filmed continuously for a further 30 min. The baseline tail temperature for each rat was averaged from three measurements every 10 s during the 30-s baseline filming. The 30-min response of the tail surface was then measured as the difference from each individual rat’s own baseline temperature (referred to as ‘difference from baseline’ hereafter). Rats were weighed once at the end of the experiment immediately after the test trial finished.

### Imaging set-up

The rats were imaged with an infra-red thermal camera (FLIR A65, f = 25 mm, spatial resolution 0.68 mrad, thermal sensitivity < 0.05°C @ +30°C, FLIR Systems, Wilsonville, OR, USA) while in the testing arena. The thermal camera was mounted on a clamp stand which was positioned 55 cm above the floor of the test arena. The rats were also videoed with a GoPro HERO 7 Silver 4K Action Camera (GoPro Inc, San Mateo, USA) attached to the top of the arena with a mount (GorillaPod 500 Action, JOBY, CA, USA) for behavioural analysis. Both cameras were positioned such that the entire test arena was within their field of view. The thermal videos were recorded at a frame rate of 30 frames per min. Air temperature and relative humidity of the room were also measured at 5-min intervals during all trials with an EasyLog USB logger (Lascar Electronics Ltd, UK) attached to the camera stand.

### Thermal data extraction

**Figure 2.**
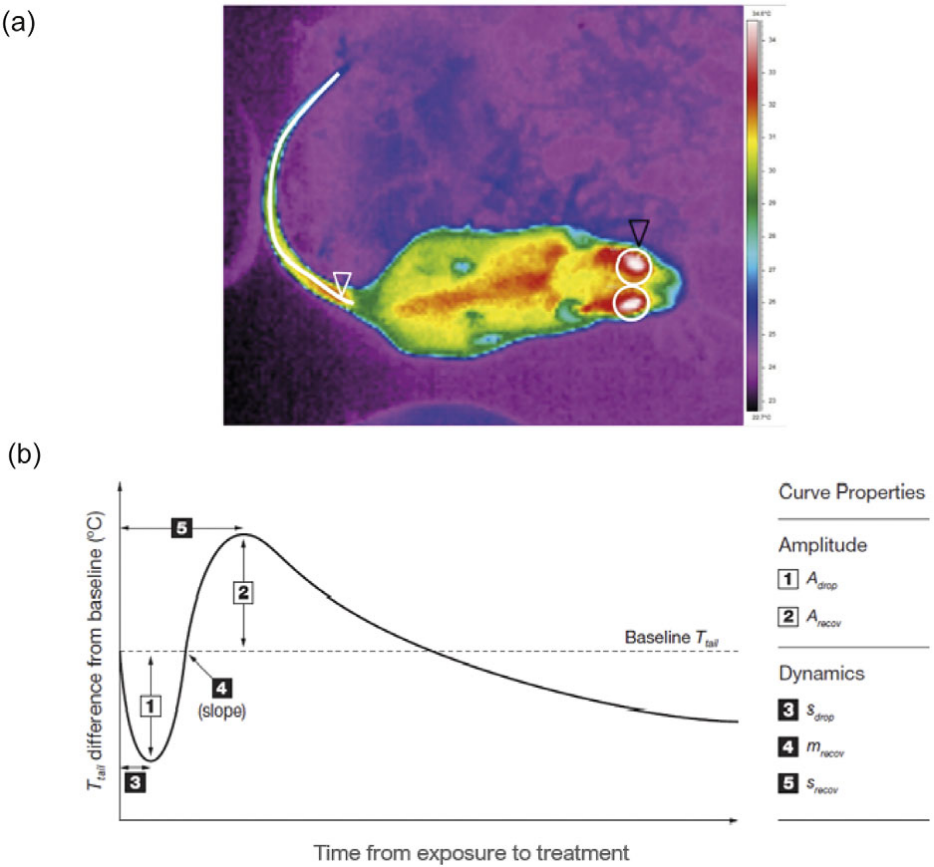
Thermal data extraction. (a) Thermal image of a rat in the testing arena viewed in the ‘rain’ (rainbow) palette in ThermaCAM Researcher software. A bendable line (white arrow) was drawn manually to extract the maximum temperature of the whole length of the tail. Left and right eye temperatures (black arrow) were also recorded and will be reported elsewhere. (b) The schematic standardised tail surface temperature response to a stimulus, identifying five distinct curve properties. The amplitude of the initial decrease in individual tail temperature from baseline (A_drop_, 1), defined as the minimum value of the temperature difference from baseline (T difference) before the first rise of temperature back towards the baseline, and the amplitude of the maximum recovery (A_recov_, 2) was defined as the highest T difference value recorded after A_drop._ The time elapsed (s) to reach A_drop_ was designated as S_drop_ (3). The rate of change of temperature from A_drop_ to A_recov_ was represented by the slope M_recov_ (4). The time elapsed (s) to reach A_recov_ was designated as S_recov_ (5).

All thermal image sequences were extracted using FLIR ThermaCAM Researcher Pro 2.10™ (FLIR Systems Inc). The tail was the ROI used as it displays the most informative body surface temperatures in rats (Wongsaengchan *et al.* 2023). The emissivity of bare skin is 0.98 (McCafferty 2007), the air temperature and relative humidity at the nearest 5 min and the distance between the object and the camera (55 cm) were inputted into the software. The accuracy of temperature readings was validated using black insulation tape attached to the EasyLog thermister (Wongsaengchan 2022). Thermal sequence images were viewed using the palette ‘rain’ (rainbow) and the most suitable thermal image (rat not sitting on its tail and their body and the head parallel with the floor) was selected every 10 s for the first 4 min after treatment exposure and every 60 s for the remaining video. For each selected thermal image, a line along the middle of the entire length of the tail was delineated manually using the ThermaCAM drawing tool ‘bendable line’ and (Figure 2[a]) eye temperature was also recorded and will be reported elsewhere. From the delineated tail we extracted the maximum temperature and then plotted the maximum temperature difference from baseline against time to produce the thermal response curve that we then compared between treatment groups and sexes (for a justification of sampling intervals and extracting maximum temperatures, see Wongsaengchan *et al.* 2023). The tail thermal curve properties (Figure 2[b]) of each individual rat were also extracted for analysis as the dynamic response also exhibited the five curve properties and these were reported to be sensitive to arousal magnitude of negative stimuli, especially the rate of recovery (Wongsaengchan *et al.* 2023). If there was new defaecation or urination visible in the thermal images as an area on the substrate that was warmer than the surrounding at that sampling time-point, this was collectively termed ‘Elimination.’

### Behavioural analysis

The ethogram we used in this study was adapted from Wongsaengchan *et al*. (2023) and mutually exclusive behaviours observed and recorded are described in the ethogram (Supplementary Table 1). Behavioural data were collected using instantaneous sampling with a scan interval of 10 s based on continuous pilot observations of the behaviour of one female rat to find the optimum sampling interval (Martin & Bateson 2007). The observer was aware of the treatment allocation as it was visible in the videos but was blinded to the sex, cage and rat ID. Proportion of scans showing each behaviour was calculated per total scans per 10 min, excluding unidentified ‘Other’, and were then grouped into four behavioural groups using Principal Component Analysis (Supplementary Figure 1) with R package; ‘FactoMineR (Lê *et al.* 2008) to reduce type I error from separate analysis of a large number of different behaviours. The four behavioural groups were: Explorative (Explore, Eat, Interact with object); Resting stationary (Rest, Stationary, Groom, Non-intake); Fear/Anxiety (Freeze, Dart); and Escape/Mobility (Escape, Rear, Wall grab, Climb, Walk). Latency to eat, the time from reward placement in the arena until the time rats started eating, was recorded for rats that received food rewards as the latency taken to approach food in a novel situation has been shown to be longer due to novelty fear or ‘bait shyness’ in anxious or stressed individuals to avoid the risks from food (Deacon 2011).

### Statistical analysis

All analyses were completed in R version 4.1.1 (R Core Team 2019). The sample size of n = 6 rats per sex per treatment (either neutral, one Cheerio or three Cheerios) was based on the sample size of a similar study of surface temperature response to different arousal levels of acute restraint stress in laboratory rats (Wongsaengchan *et al.* 2023) calculated using 80% power and 0.9 smallest standardised effect size at the 5% significance level. One female rat which jumped out from the testing arena continuously for more than 10 min and one female rat that did not approach the food reward were excluded from the analyses as the first exhibited obvious stress and the second did not provide comparable data. Therefore, the female neutral and 3-Cheerios groups had only n = 5 while the other treatment groups had n = 6. The mean difference in each rat’s maximum tail temperature from their own baseline temperature for each time-point and the five tail thermal curve properties of each rat were used as response variables. These were analysed separately using general linear mixed models (GLMMs) with the ‘nlme’ package in R (Pinheiro *et al.* 2018) with treatment, sex, time-point as a quadratic term to capture a non-linear relationship, time of day (TOD), environmental factors that can affect measured infra-red radiation (i.e. air temperature, humidity), animal factors that can affect measured infra-red radiation (i.e. location in the arena, posture, body mass) and possible interactions as explanatory variables and animal identity (Rat ID) as a random factor (see Supplementary Table 2). Behaviours were pooled into three time-blocks of 10 min each and the proportion of scans per 10 min showing each behavioural group used as response variables. ‘Elimination’ behaviour was observed from thermal images at a different interval than other behaviours and treated as counts per 10 min and analysed with a Poisson residual distribution. Correlations between behavioural response and thermal response were examined using GLMM and multivariate exploratory data analysis (see Supplementary Figure 3).

In all statistical models, non-significant terms were removed with backward-stepwise model simplification using the likelihood ratio test (LRT) at a significance level of 0.05. The *post hoc* test in the R package ‘lsmeans’ (Lenth 2016) was used to further examine statistically significant differences between groups where appropriate. Model assumptions were diagnosed with graphical tools and functions in R (Zuur *et al.* 2010) and the response variables were log- or square-root transformed where necessary to meet the normality of residuals, independence of residuals, co-linearity and homogeneity of variance assumptions suitable for GLMMs testing. When checking explanatory variables for co-linearity, body mass and sex were positively correlated (*r* = 0.80, df = 32) and had a high variance inflation factor (VIF = 8.83), therefore, ‘body mass’ was excluded from models that included ‘sex’ and was only included in models analysing male or female rats separately. The GLMMs models of tail temperature were examined for temporal autocorrelation using the ‘acf’ and ‘pacf’ plotting functions in R showed a significant autocorrelation at a lag of 1 and much lower spikes for the subsequent lags. Thus, the correlation correction ‘corAR1()’ was added to tail temperature GLMM models.

## Results

### Body surface temperature

**Figure 3.**
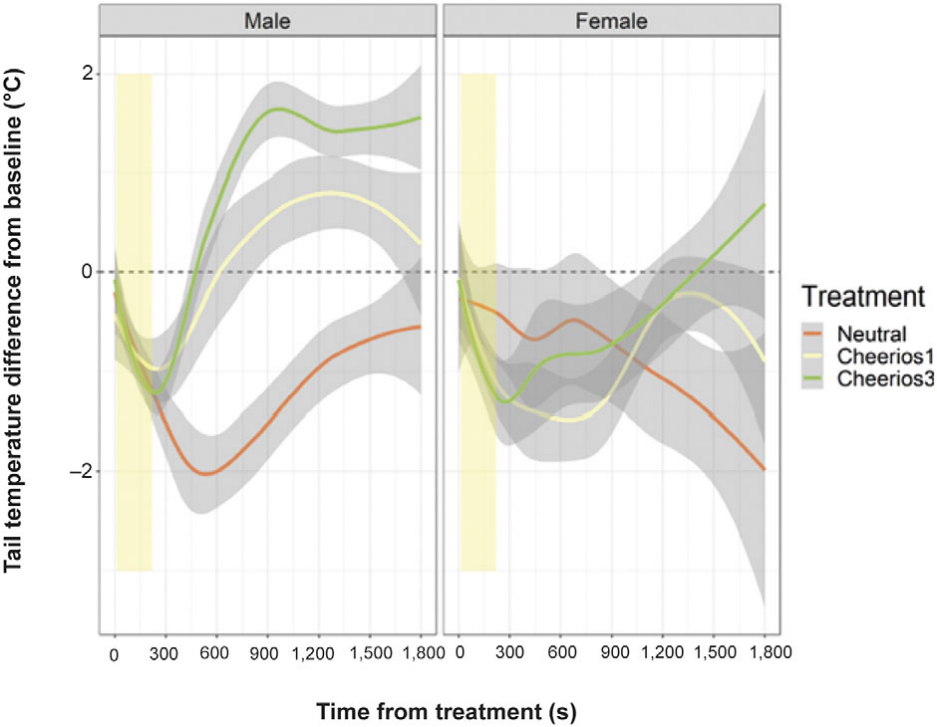
Sex difference in tail temperature response to food reward with different magnitudes.The figure shows spline-fitted lines and 95% confidence interval (grey bands) of the maximum tail temperature responses of rats to either no reward (neutral), one Cheerio or three Cheerios (n = 6 for each response curve except female neutral and female 3-Cheerios groups have n = 5). The thermal response of the tail shown in the graph was of the rats being exposed to the treatment until 30 min post-treatment. The baseline temperature (dashed line) was calculated from three measurements every 10 s of the 30 s baseline filming immediately before treatment exposure. The yellow bands represent the range in reward consumption.

Female rats had lower baseline tail temperatures (30.36 [± 1.70]°C, n = 16) than male rats (32.41 [± 0.92]°C, n = 18; LRT, 2∆LL = 82.93, df = 1; *P* < 0.001). After exposure to treatments, tail temperature initially decreased from the baseline in all groups and subsequently increased back towards baseline temperature and overshot the baseline (rewarded males and females) or continued to decrease (neutral females) (Figure 3). The tail thermal response curve was non-linear and differed between the three treatments (LRT, treatment-by-time: 2∆LL = 12.26, df = 2; *P* = 0.002; treatment-by-time^2^: 2∆LL = 14.44, df = 2; *P* < 0.001) and between the two sexes (LRT, sex-by-time: 2∆LL = 37.06, df = 1; *P* < 0.001; sex-by-time^2^: 2∆LL = 22.58, df = 1; *P* < 0.001; Supplementary Table 2).

The tail temperature difference from baseline over time differed between the three treatments in both male and female rats (male, treatment-by-time: 2∆LL = 25.55, df = 2; *P* < 0.001, treatment-by-time^2^: 2∆LL = 20.23, df = 2; *P* < 0.001, female, treatment-by-time: 2∆LL = 18.11, df = 2; *P* < 0.001, treatment-by-time^2^: 2∆LL = 17.29, df = 2; *P* < 0.001; Supplementary Table 2). The tail cooled the most, started to recover later and remained below the baseline during the 30 min in males in the neutral group compared to the rewarded males. Tail temperature, however, changed similarly over time in the two rewarded groups, initially cooling and later overshooting the baseline, although the tail temperature of male rats rewarded with three Cheerios remained above baseline for longer than in males rewarded with one Cheerio (Figure 3). In females, the tail cooled initially and returned towards baseline but did not overshot above baseline during the 30 min of recording except the 3-Cheerios rewarded group (Figure 3). A separate GLMMs of only the food reward groups showed no significant difference in the tail thermal response between rats rewarded with one or three Cheerios (LRT, 2∆LL = 1.18, df = 1; *P* = 0.278) but between sexes where females showed greater decrease of tail temperature after exposure to rewards and lower recovery than males (LRT, 2∆LL = 31.42, df = 2; *P* < 0.001; Figure 3). Since tail temperature response over time differed between sexes in all models, each sex was then analysed separately with GLMMs (Supplementary Table 2). Furthermore, the tail temperature response in females differed between postures (Supplementary Table 2) where the tail temperature was cooler when female rats were inactive compared to when walking (LSM; *P* = 0.008) and eating (LSM; *P* = 0.041), but there were no significant differences or trends with other postures. The tail temperature response was not related to body mass, location, air temperature, humidity and time of day (see Supplementary Table 2).

**Figure 4.**
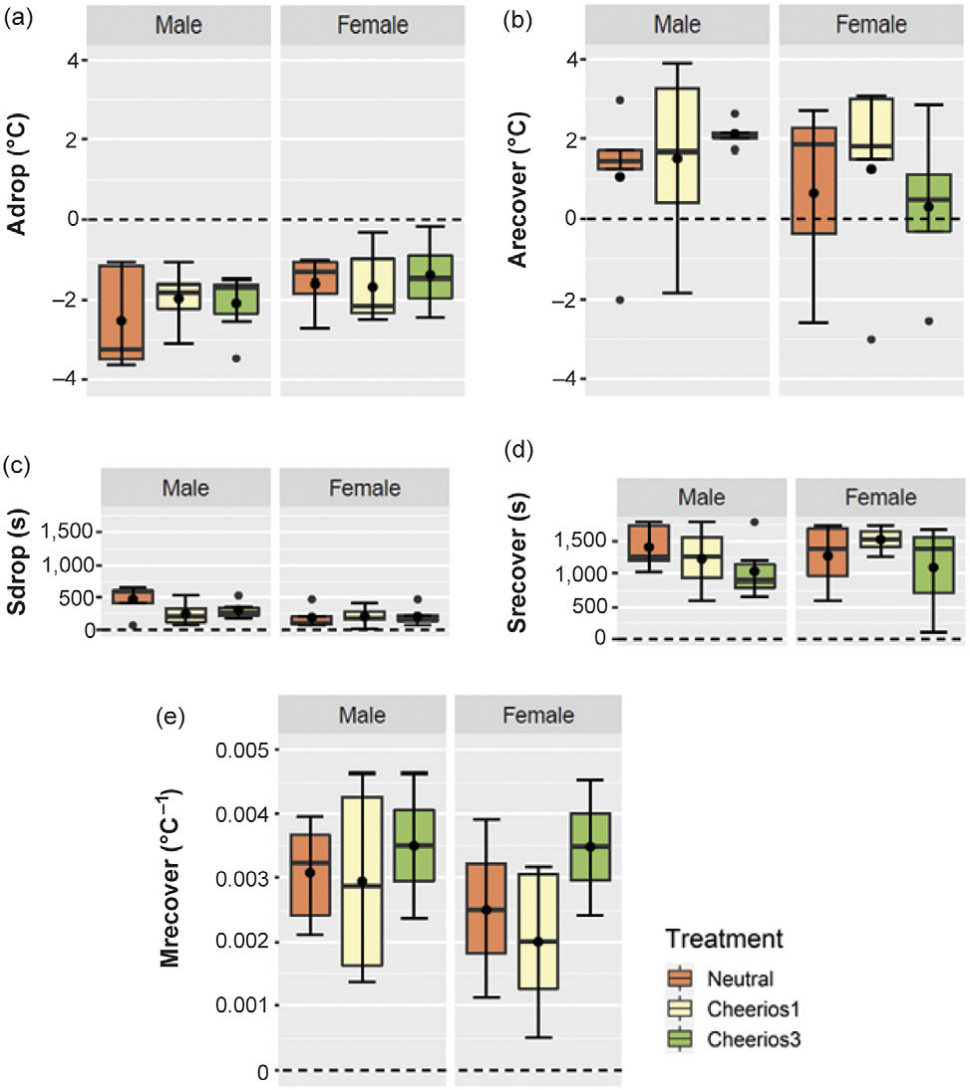
Boxplots display the distribution of the amplitudes and the dynamics of specific properties of the tail temperature response to 0 (neutral), 1 and 3 Cheerios according to sex. The median for each dataset (n = 6 per box except female neutral and female 3-Cheerios groups have n = 5) is indicated by the black centre line, and the lower and upper hinges of the box are the inter-quartile range (IQR). The extreme values (within 1.5 times the IQR from the upper or lower quartile) are the ends of the lines extending from the IQR. Outliers are represented as filled circles outside the whiskers and whiskers are the standard deviations. The specific thermal response properties plotted are the amplitude of the drop of the temperature (A_drop_: a) and the rise of the temperature (A_recover_: b) and the time taken to reach A_drop_ (S_drop_: c) and the time taken to reach A_recover_ (S_recover:_ d) (s). The rate of change of temperature from A_drop_ to A_recover_ was represented by the slope (M_recover_: e).

To further investigate how the tail temperature response differed between the treatments and sexes, five specific components of the change in tail temperature (Figure 2[b]) were identified for each individual rat and analysed separately (Figure 4). Due to the non-significant treatment by sex interaction (Table 1), the specific curve components of the change in tail temperature were not analysed for each sex separately. Most of the curve components of the tail thermal response did not differ between treatments and between sexes (Table 1). Only the rate of recovery (M_recov_) differed between the three treatments (Table 1). *Post hoc* analysis showed that rats exposed to three Cheerios recovered faster than rats in the neutral groups (LSM; *P* =0.033) and a trend that rats exposed to three Cheerios recovered faster than rats exposed to one Cheerio (LSM; *P* = 0.054). Only the time to reach the lowest tail temperature (S_drop_) differed between the two sexes (Table 1), where males took more time to reach the lowest drop of tail temperature than females. None of the specific components of the change in tail temperature were related to air temperature, humidity and time of day.

**Table 1.** GLMM analysis of the amplitudes and the dynamics of specific properties of the tail temperature response to either no Cheerios, one Cheerio or three Cheerios (n = 35). The table shows the fixed effects included in the models. Individual rat identification is the random effect also included in the models but is not shown. The significant *P*-values of using log likelihood ratio tests are shown in bold italic font

Tail thermal curve properties	Analysis term (fixed effect)								
	Treatment			Sex			Sex×Treatment		
	2∆LL	df	*P*-value	2∆LL	df	*P*-value	2∆LL	df	*P*-value
A_drop_	0.43	2	0.806	1.99	1	0.158	0.58	2	0.747
A_recov_	2.39	2	0.302	3.69	1	0.055	0.07	2	0.968
S_drop_	2.55	2	0.28	4.15	1	***0.041***	3.45	2	0.178
S_recov_	3.61	2	0.165	0.38	1	0.538	1.42	2	0.492
M_recov_	8.34	2	***0.015***	2.67	1	0.102	1.54	2	0.464

The specific thermal response curve properties are the amplitude of the drop of the temperature (A_drop_) and the rise of the temperature (A_recover_) and the time taken to reach A_drop_ (S_drop_) and the time taken to reach A_recover_ (S_recover_) in seconds. The rate of change of temperature from A_drop_ to A_recover_ was represented by the slope (M_recover_).

### Behavioural response

**Figure 5.**
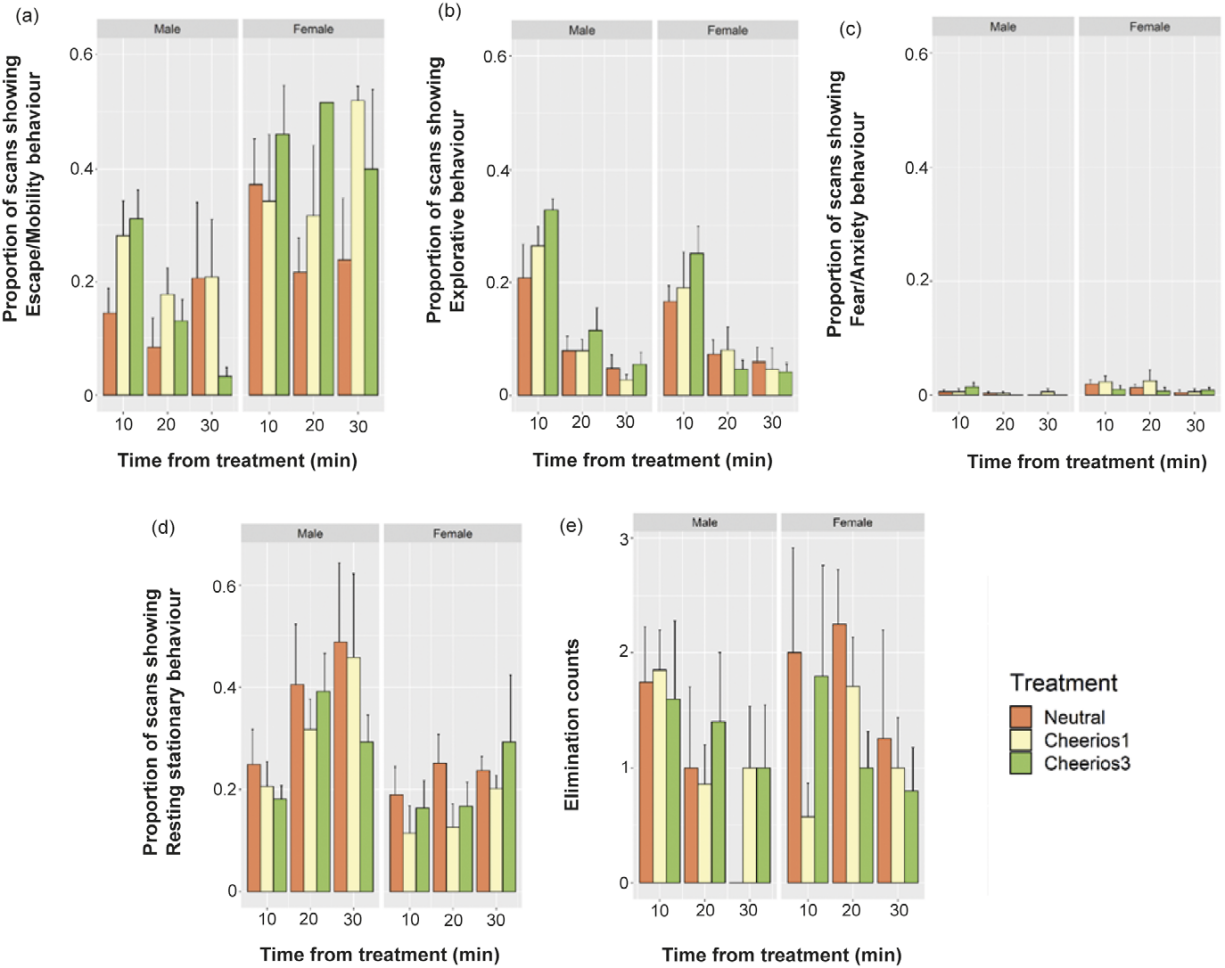
Bar graphs indicating mean (± SE) of proportion of scans showing (a) ‘Escape/Mobility’, (b) ‘Explorative’, (c) ‘Fear/Anxiety’ and (d) ‘Resting stationary’ behaviours and eliminating (defaecating/urinating) behaviour counts per 10 min of rats during the 30-min time-period after treatment (neutral, one Cheerio or three Cheerios) exposure (n = 34).

The most performed behaviours by rats in this study were ‘Rest’, ‘Stationary’, ‘Wall grab’ and ‘Groom’ while the least performed behaviours were ‘Non-intake’, ‘Freeze’ and ‘Dart’ (Supplementary Figure 2). After behaviours were pooled into four groups (see *Materials and methods*: *Behavioural analysis*), the ‘Explorative’ and ‘Resting stationary’ behavioural groups were affected by treatment depending on time block (Table 2). ‘Explorative’ behaviours increased, while ‘Resting stationary’ behaviours decreased with a greater number of Cheerios, however, the difference with the neutral group disappeared over time (Figure 5[b], [d], Table 2). The two sexes behaved differently after exposure to treatments (Table 2): female rats performed more ‘Fear/Anxiety’ and ‘Escape/Mobility’ behaviours than male rats while male rats were exhibiting ‘Resting stationary’ behaviours more than female counterparts (Figure 5), but the sexes did not respond differently to reward. ‘Escape/Mobility’, ‘Fear/Anxiety’ and ‘Elimination’ behaviours decreased over time while changes over time in ‘Resting stationary’ and ‘Explorative’ behaviours differed between treatments (Table 2, Figure 5). The latency to eat ranged from 8–201 s (mean [± SE]: 42.54 [± 44.84]) after the food reward was introduced. Furthermore, longer latency to eat was associated with lower baseline temperature (LRT, 2∆LL = 6.43, df = 1; *P* = 0.011). PCA analysis was also performed to explore correlation between behaviours and body surface temperature (baseline tail temperature and rate of recovery of the tail temperature response curve; Supplementary Figure 3). Rats that performed more ‘Resting stationary’ behaviour were those with higher baseline tail temperature and later tail cooling while baseline temperature was negatively correlated with ‘Escape/Mobility’ behaviours. The rate of recovery of tail temperature (M_recover)_ was negatively correlated with both ‘Explorative’ and ‘Fear/Anxiety’ behavioural groups.

**Table 2.** GLMM analysis of proportion of scans showing rat behaviours per 10 min during the 30-min filming after exposure to either no Cheerios, one Cheerio or three Cheerios (n = 35). The table shows the fixed effects included in the models. Individual rat identification is the random effect also included in the models but is not shown. The significant *P*-values of using log likelihood ratio tests are shown in bold italic font

Behavioural group	Analysis term (fixed effect)																	
	Treatment			Sex			Time			Sex×Treatment			Time×Treatment			Time×Sex		
	2∆LL	df	*P*-value	2∆LL	df	*P*-value	2∆LL	df	*P*-value	2∆LL	df	*P*-value	2∆LL	df	*P*-value	2∆LL	df	*P*-value
Elimination	0.54	2	0.7650	0.30	1	0.5838	5.54	1	***0.0185***	3.73	2	0.1551	1.91	2	0.3856	2.57	1	0.1092
Escape/Mobility	2.11	2	0.3484	12.68	1	***0.0004***	2.36	1	0.1248	2.05	2	0.3595	5.19	2	0.0746	1.76	1	0.1847
Explorative				3.15	1	0.0761				0.84	2	0.6572	6.19	2	***0.0453***	2.30	1	0.1297
Fear/Anxiety	0.78	2	0.6760	8.71	1	***0.0032***	6.56	1	***0.0104***	1.20	2	0.5500	0.51	2	0.7764	0.03	1	0.8623
Resting stationary				15.26	1	***0.0001***				0.74	2	0.6919	7.18	2	*0.0276*	2.03	1	0.1545

## Discussion

This study demonstrates, for the first time in rats, that the dynamic response of body surface temperature measured with IRT differs between rats with and without food reward and between the sexes. These effects were supported by the concurrent approach-avoidance behavioural response. The tail surface temperature decreased after exposure to food reward. Extracting individual curve properties of the tail temperature response gave insights that the treatment difference was seen in the rate of the recovery (M_recover_). The tail temperature of rats given the larger food reward recovered faster than rats without food reward and tail temperature of male rats took more time to reach the minimum temperature (S_drop_) than in female rats. However, the overall tail temperature response did not differ over time between the small and larger food reward groups.

The first objective of this study was to determine if IRT could indicate positive affective state (valence). We found that IRT could differentiate a rewarding experience from a neutral condition but not between the two magnitudes of food reward. After exposure to a food reward, the tail surface temperature decreased with the same direction of thermal change after an exposure to the neutral treatment as well as negative events such as acute restraint (Wongsaengchan *et al.* 2023) and foot shock (Vianna & Carrive 2005). These findings are in accordance with previous work in non-human primates (Ioannou *et al.* 2015; Kano *et al.* 2016; Chotard *et al.* 2018; Heintz *et al.* 2019), cows (Proctor & Carder 2015, 2016) and hens (Moe *et al.* 2012) that positive emotional state may have the same effect on the peripheral temperatures of mammals as a negative state does. Our results suggest that the arrival and subsequent consumption of food reward elicited a positive, moderate arousal state such as excitement or reward anticipation (Gygax *et al.* 2013) as opposed to stimulus novelty since it has been ruled out by two exposures of these same rewards in the home cage (see Habituation phase). Similar effects have been found in chickens where the comb temperature drops in response to the conditioned positive anticipation and delivery of a favoured food (Moe *et al.* 2012). These findings suggest that the SIH phenomenon of the sympathetic activation may be influenced by both positive and negative emotional states. The positive emotion induced by feeding, however, would have involved parasympathetic co-activation and would have facilitated the recovery of the thermal response in this study. Whether a relation between emotion and the organisation of ANS activity exists is not well known and needs to be investigated with future works employing several ANS parameters including cardiovascular, electrodermal and respiratory measures. In our previous IRT study (Wongsaengchan *et al.* 2023), we found a slight increase in eye temperature after restraint, and after food reward in this study, but the results were not correlated with tail temperature response, corticosterone level and behaviours. Furthermore, the asymmetrical response of nostril temperature was reported to potentially reflect valence of emotions in dogs (Telkänranta 2016), according to the emotional lateralisation theory (Leliveld *et al.* 2013). Although one more possibility for assessing valence using body surface temperature could lie in the lateralised temperature response due to emotional cerebral and behavioural lateralisation (Rogers 2010; Leliveld *et al.* 2013, Goursot *et al.* 2021), we did not find valence clues from changes in eye temperature in our previous stress study in rats.

The second objective of this study was to assess whether body surface temperature measured with IRT of male and female rats can also quantify increasing levels of reward magnitude (arousal). IRT was reported in rats (Wongsaengchan *et al.* 2023) and in hens (Herborn *et al.* 2015) to allow quantification of stress from mild and brief restraints of different magnitude, however, we were unable to quantify positive affect from one and three food reward items used in this study. Possibly, the rewards used to induce positive affective states in this study were not sufficiently different in magnitude to be detectable by IRT. The degree of temperature change will depend on sympathetic nervous activation which may even lead to an activation of the hypothalamic-pituitary-adrenal (HPA) axis and an increase in plasma corticosterone levels (Lowe *et al.* 2005; Stewart *et al.* 2005; Ouyang *et al.* 2021). The reward magnitude difference in this study was based on a previous work measuring running speed in rats using a forced-choice maze paradigm where three Cheerios clumped horizontally together would induce a greater positive affect due to greater density and surface area (Wadhera *et al.* 2018). At the very least, the larger reward increased the duration of the positive event during imaging. This also raises questions regarding the triggers for SIH, which is often considered to be associated only with negative states and high arousal (Bouwknecht *et al.* 2007; Kuraoka & Nakamura 2011). In addition, in laboratory rodents, most measures are reported to capture the extremes of the scale from negative to positive valence and there is a lack of established welfare measures for the range between neutral and positive valence (Jirkof *et al.* 2019). This should be investigated further using more pleasant stimuli or spontaneous positive events (e.g., play) with a greater difference in magnitude. Transparent, infra-red materials and add-on equipment have been developed recently and can be made into a temporary lid or attached to a home cage to create an infra-red inspection window (e.g., IR Material Window, Edmund Optics Inc, Barrington, USA; FLIR IR WINDOWS or FLIR IRW-XPC/XPS 2020© Teledyne FLIR, OR, USA). This may therefore allow simultaneous recording of surface temperature with other physiological and behavioural measurements in a home cage that may be useful for examining dynamics of positive affective states in future studies.

As we avoided measuring physiological welfare markers, especially invasive procedures which would confound the results, we only have the concurrent behavioural responses to validate our thermal responses. However, our behavioural analysis showed that, unlike behavioural response to acute stress in previous studies (Gregus *et al.* 2005; Brenes *et al.* 2009; Wright *et al.* 2013; Jaisinghani & Rosenkranz 2015; Wongsaengchan *et al.* 2023), rats in this study performed mostly resting and stationary (not freezing/immobility) behaviours and exhibited substantially fewer anxiety/fear behaviours such as freezing and darting (Rodgers 1997; Vianna & Carrive 2005; Barnum *et al.* 2007; Nikaido & Nakashima 2009; Brenes *et al.* 2009; Barker *et al.* 2010; Seffer *et al.* 2014; Gruene *et al.* 2015; Magara *et al.* 2015; Le Moëne & Ågmo 2018) (Supplementary Figure 2). This suggests that our experiment was not as stressful for the rats compared to other previous stress studies. Observations of behaviour indicating fear/anxiety still existed, even minimally, in this study of positive stimuli potentially because of the use of the open-field-like test arena for thermal image recording, although rats have gone through the habituation protocol to minimise effects of isolation in the test arena, the experiment lasting 30 min in the test arena may have become an ongoing mild stressor after the food reward was eaten.

Nevertheless, we found a food reward effect in the behavioural response, supporting the different thermal responses between rewarded and non-rewarded rats. The most performed resting and explorative behaviours differed between reward treatments depending on time-point. Explorative behaviours increased while resting stationary behaviours decreased with a greater number of food rewards, suggesting rat anticipation after food rewards exposure. Some rats were also seen looking for more rewards after they finished eating (CW personal observation 2020). Anticipation reflects the activation of the reward system and was one of the most documented behavioural measures for positive affective states which rats can express alone, as opposed to play or affiliative behaviours when in groups (Boissy *et al.* 2007; Makowska & Weary 2013). However, most studies observing anticipation use trained rats with regular presentation of rewards for rats to learn a condition that rats would expect reward and show anticipating behaviours (Van der Harst & Spruijt 2007; Zimmerman *et al.* 2011; Anderson *et al.* 2020). This study, on the other hand, only presented food reward once in the arena and some rats might not have expected that more food reward would be given. The explorative behaviours increased with the number of Cheerios during the first 10 min, possibly because the rats were less stressed by the arena due to the counter-effect of Cheerios in a dose-dependent manner. In humans, reward signalling in the brain was shown to be attenuated by aversive counter-conditioning (Kaag *et al.* 2016). Therefore, once the positive reward ceased, rats in this study reduced explorative behaviours over time compared to the unrewarded rats and instead rested more. The positive effect of Cheerios could be from the quality (visual appearance) and quantity (more surface area) of zero, one and three Cheerios once the rats saw their reward (Wadhera *et al.* 2018). The difference of the magnitude, however, was only strong enough to yield significant LSM *post hoc* comparisons between the neutral and the three-Cheerios group. This indicates that our treatments did not differ enough in reward magnitude, in agreement with the thermal responses.

Behavioural responses were analysed in relation to the thermal results and ‘Resting stationary’ behaviours were associated with higher baseline tail temperature and a more delayed tail cooling while baseline tail temperature was negatively correlated with escape behaviours. These results suggest that a lower baseline reflected a more anxious individual as was shown in small birds that eye surface temperature was also negatively correlated with baseline circulating glucocorticoid levels (Jerem *et al.* 2018). The rate of recovery of the tail temperature was found to be faster in rats that showed less fear/anxiety behaviours and, surprisingly, less explorative behaviours. However, the vectors of these variables covered only a few individual rats (Supplementary Figure 3), making the interpretation of these variables difficult. Furthermore, longer latency to eat was associated with lower baseline tail temperature. In anxious or stressed individuals, latency to approach food in a novel situation is longer due to neophobia or ‘bait shyness’ which is often used as a way of measuring anxiety (Deacon 2011). Therefore, the behavioural responses in this study suggested that rats expressed mostly non-stress behaviours after provision of a food reward and that a lower baseline and a longer latency to eat may be helpful in identifying more anxious individuals. Although most of the variation in the latency to eat was explained by baseline tail temperature of rats, different subjective experience of the prior exposures to food reward (two occasions in the habituation phase) between rats within the same cage could also contribute to some of the variation (Campbell *et al.* 1972; Galtress & Kirkpatrick 2010). ‘Elimination’ (i.e. defaecation and urination) is a proxy measure of fear/anxiety which should be positively correlated with anxiety level and negatively correlated with locomotor activity (Wallace & Rosen 2000; Brenes *et al.* 2009; Bowen *et al.* 2012; Wright *et al.* 2013), could only be seen when rats were moving away from their faeces and urine or stood on their rear legs and was positively correlated with ‘Escape/Mobility’ behaviours, indicating that ‘Eliminate’ counts therefore effectively measured movement. The time effect could then also be explained that rats overall moved more initially after exposure to treatment and then reduced movement over time.

As in a previous stress experiment in rats (Wongsaengchan *et al.* 2023), female rats responded with a more prolonged duration than male rats. However, female rats given food rewards recovered better than female rats in the neutral group and after 1-min restraint (Wongsaengchan *et al.* 2023), suggesting that these Cheerios may be more rewarding to females than to males. Previous research has also suggested stronger preference for sweet reward in female compared to male rats (Valenstein *et al.* 1967; Sclafani *et al.* 1987) and that adult females exhibited greater initial consumption rates and stronger magnitude of neural responsiveness to high sugar food reward than adult males (Marshall *et al.* 2017; Sinclair *et al.* 2017). Sex differences in opioid and dopaminergic signalling and autonomic nervous control of the cardiovascular system both prior to and during food intake may contribute to the enhanced responses to food reward in females (Gruene *et al.* 2015; Sinclair *et al.* 2017). The more rats performed resting behaviours, the higher the baseline tail temperature. On the other hand, the lower the baseline temperature, the more escaping behaviours and longer latencies to eat were seen. The sex difference in tail baseline surface temperature also suggested that female rats were more anxious than male rats, with the SIH process already having started with tail cooling apparent even before testing while the eye temperature which was related to the core temperature (Kessel *et al.* 2010) remained similar to the males (eye results will be reported elsewhere). Sex differences in the behavioural response was also found in this as well as in previous studies (Campbell *et al.* 2003; Dalla *et al.* 2011; Colom-Lapetina *et al.* 2019; Knight *et al.* 2021). Fear/anxiety-related behaviours were performed more by female than male rats whereas resting/stationary behaviours were performed more in male than female counterparts, but this was not affected by the treatments. The lack of sex-by-time interaction in this study could suggest that the sexes differed in their behavioural responses depending on the valence of stimuli driven by a fundamental difference in stress/reward processing (Mashoodh *et al.* 2008; Marshall *et al.* 2017; Chowdhury *et al.* 2019). The main sex effect shown in this study, however, mainly reflected the different strategies to cope with the testing arena between the sexes reported in previous studies (Keating 2010; Gruene *et al.* 2015; Bangasser & Wicks 2017; Le Moëne & Ågmo 2018). Several theories have been proposed to explain the cause of sex differences (Tamres *et al.* 2002; Luine & Dohanich 2008; Bangasser & Wicks 2017). For example, in the natural selection theory, different ethological demands and biological support of evolutionary explanations render that females may be more likely to survive a threat if they are active and able to detect and escape it, while males may benefit more by conserving energy and using more passive strategies (Jonasson 2005; Colom-Lapetina *et al.* 2019).

The potential confounding factors in the present study were any physical activities that trigger the sympathetic nervous activation such as walking (Pavlidis *et al.* 2000), food consumption (van Baak 2008; Ioannou *et al.* 2015) as well as the environmental temperature (Fernández-Cuevas *et al.* 2015; Tattersall 2016; Nord & Folkow 2019), humidity (Fernández-Cuevas *et al.* 2015; Tattersall 2016) and time of day (Koch *et al.* 2017; Oka 2018) that the test was conducted. These were all included in analysis and only posture was found to affect body surface temperature. The tail temperature was cooler when female rats were inactive compared to when eating. Increased metabolism during eating produces extra heat and could be an explanation for this finding (van Baak 2008; Ioannou *et al.* 2015).

### Animal welfare implications

Advancing our knowledge of positive welfare state in non-human animals is not only important for improving the welfare of animals but that of human caregivers and the quality of animal research. The thermal curve component analysis of the tail has revealed the ability of IRT to non-invasively compare valence between neutral and positive state relatively in rats of both sexes, but the ability of IRT to quantify positive states induced by different reward magnitudes was not supported. This could potentially be due to the rewards used in this study not differing sufficiently in their magnitude. Although tail temperature might not be able to reveal valence in discrete emotion terms due to the overall shape and direction of the tail thermal curve response being very similar during both negative and positive experiences, it can tell which event is relatively more positive than another by showing a higher rate of recovery. This could then be used to assess the continuum between negative and positive affective states that is part of the dynamic welfare of an animal. The validation of a surface temperature approach to assess, not only negative, but also positive events and their magnitude gives the possibility to provide a non-invasive, real-time, continuous means by which to assess dynamic welfare, contributing to refinement of research (3Rs: replacement, reduction, refinement). Furthermore, if home cage thermal imaging can be undertaken, this approach could also be used in rodent husbandry to monitor welfare throughout life. In addition, rats in this study appeared to recover better when receiving a food reward at the start of the procedure. The food reward could be used more often before or after procedures to encourage participation and recovery.

## Conclusion

This study aimed to explore the use of IRT to measure the two dimensions of affective state (valence and arousal) to identify and quantify reward-induced positive affective state. The overall shape and direction of the tail temperature response curve cannot differentiate between valences as tail temperature dropped after exposure to neutral situation and rewards as it did when exposed to negative experiences (Wongsaengchan *et al.* 2023). However, the temperature response curve dynamic was still able to differentiate between neutral and reward-induced positive states; a higher rate of recovery (M_recov_) was found in rewarded rats as compared to unrewarded rats but was unable to quantify differences in reward magnitudes. Behavioural responses supported these thermal responses as explorative behaviours increased in the rewarded group compared to the neutral group in both sexes but did not differ between reward magnitudes. Sex differences were found in both thermal and behavioural measures, emphasising the need to consider both sexes in welfare research. To further explore the positive effects of reward and its magnitude on thermal and behavioural responses, we recommend firstly, to use stronger rewards which differ largely in their magnitude and secondly, to use non-invasive standardised tests avoiding confounding negative stimulation by thermal imaging in home cages using a permeable window to infra-red radiation. Future work could also explore the effects of sex, oestrus cycle, genetic strain, light/dark-phase, and age on surface temperature responses to negative and positive stimuli as well as to expand IRT applications to other endodermic species both in captive and wild contexts. This study contributes to our understanding of the dynamics of positive emotions induced by food reward and their effects on peripheral body temperature and behaviour.

## Supporting information

Wongsaengchan et al. supplementary material 1 Wongsaengchan et al. supplementary material

Wongsaengchan et al. supplementary material 2 Wongsaengchan et al. supplementary material

Wongsaengchan et al. supplementary material 3 Wongsaengchan et al. supplementary material

Wongsaengchan et al. supplementary material 4 Wongsaengchan et al. supplementary material

Wongsaengchan et al. supplementary material 5 Wongsaengchan et al. supplementary material

## Data Availability

Data are available at Mendeley Data: https://doi.org/10.17632/c6bm6ywnmv.1.

## Supplementary material

The supplementary material for this article can be found at http://doi.org/10.1017/awf.2023.87.
