# Supplementary material for: Non-invasive assessment of positive affective state using infra-red thermography in rats
Source: Anim Welf. 2023 Sep 29;32:e66. doi: 10.1017/awf.2023.87 (PMC10951672; doi:10.1017/awf.2023.87)
Supplement: Wongsaengchan et al. supplementary material 1 — Wongsaengchan et al. supplementary material [file S0962728623000878sup001.pdf]

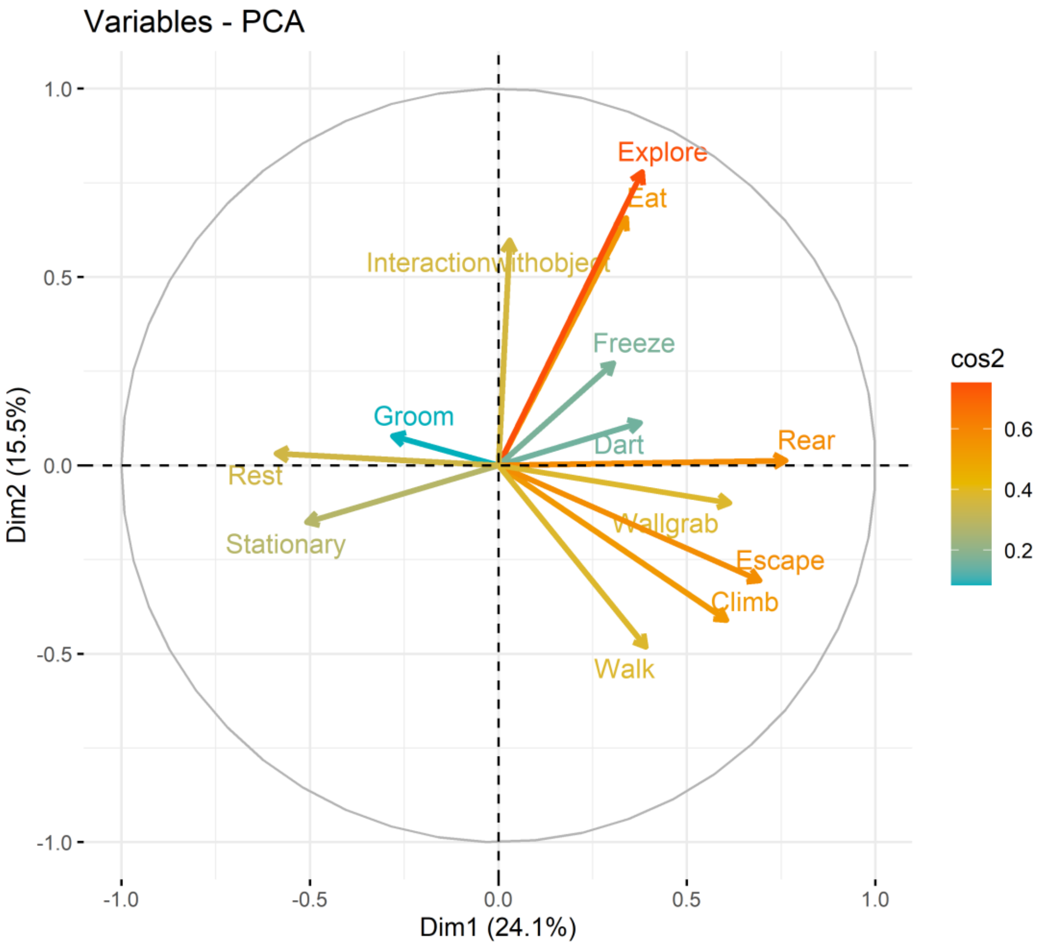

Multivariate exploratory data analysis of behaviours of rats (n=35) after exposure to 0,1 and 3 Cheerios.

A total of 15 behaviours, excluding unidentified ‘Other’ was analysed with principal component analysis (PCA) and behaviours were pooled into four functional groups for GLMM analysis. The four behavioural groups are: Explorative (Explore, Eat, Interact with object), Resting stationary (Rest, Stationary, Groom, Non-intake), Fear/Anxiety (Freeze, Dart) and Escape/Mobility (Escape, Rear, Wall grab, Climb, Walk) behavioural groups. The quality of representation for each individual was calculated as the squared coordinates (cos2). Positively correlated behaviours are grouped together. Negatively correlated behaviours are positioned on opposite sides of the plot origin (opposed quadrants).
