# Supplementary material for: Non-invasive assessment of positive affective state using infra-red thermography in rats
Source: Anim Welf. 2023 Sep 29;32:e66. doi: 10.1017/awf.2023.87 (PMC10951672; doi:10.1017/awf.2023.87)
Supplement: Wongsaengchan et al. supplementary material 2 — Wongsaengchan et al. supplementary material [file S0962728623000878sup002.pdf]

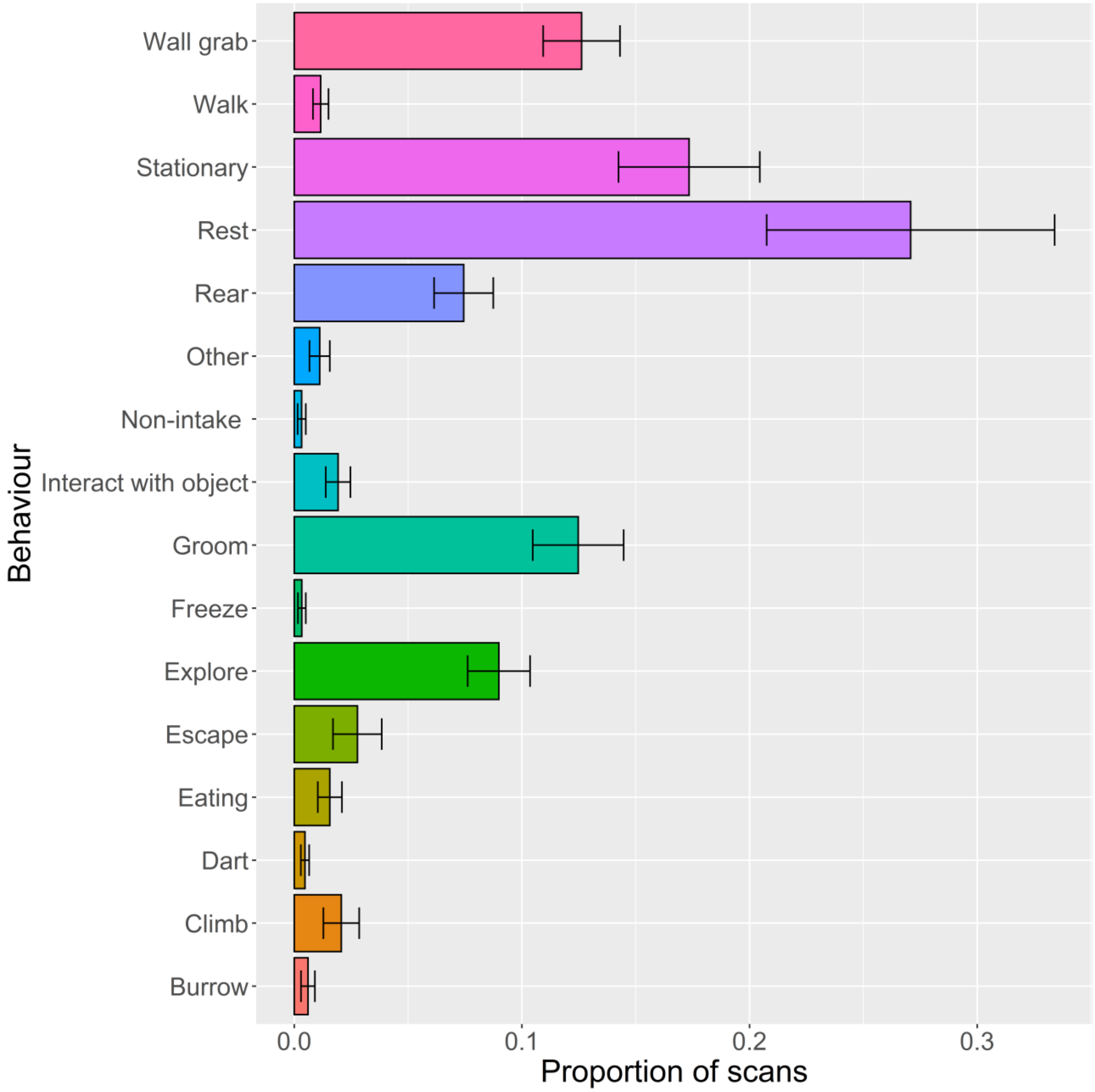

Bar graphs indicated mean ± SE of proportion of scans showing the behaviour of rats during the 30-minute time period after treatment (neutral, one Cheerios or three Cheerios) exposure (n=34).
