# Supplementary material for: Non-invasive assessment of positive affective state using infra-red thermography in rats
Source: Anim Welf. 2023 Sep 29;32:e66. doi: 10.1017/awf.2023.87 (PMC10951672; doi:10.1017/awf.2023.87)
Supplement: Wongsaengchan et al. supplementary material 3 — Wongsaengchan et al. supplementary material [file S0962728623000878sup003.pdf]

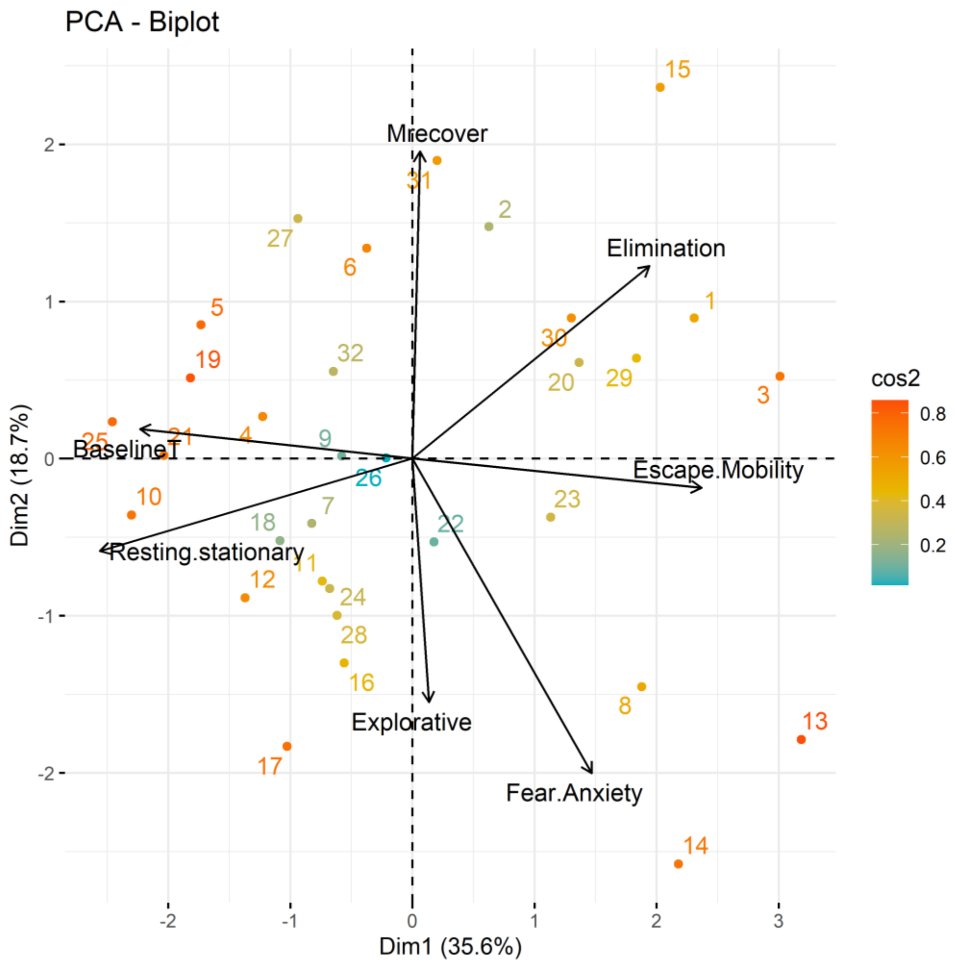

Multivariate exploratory data analysis of behavioural and tail temperature measures of rats (n=34) after exposed to either zero, one and three Cheerios.

The quality of representation for each individual was calculated as the squared coordinates (cos^2). The rate of change of tail temperature from the lowest drop to the highest recovery was represented by the slope: $M_{recover}$. Baseline temperature (BaselineT) was averaged from three measurements of tail temperature 30 seconds before treatment exposure. Positively correlated variables are grouped together. Negatively correlated variables are positioned on opposite sides of the plot origin (opposing quadrants). The length of the vector represents the quality of the behaviours on the factor map.
