# Supplementary material for: Non-invasive assessment of positive affective state using infra-red thermography in rats
Source: Anim Welf. 2023 Sep 29;32:e66. doi: 10.1017/awf.2023.87 (PMC10951672; doi:10.1017/awf.2023.87)
Supplement: Wongsaengchan et al. supplementary material 4 — Wongsaengchan et al. supplementary material [file S0962728623000878sup004.pdf]

Table 1. Ethogram of rat behaviours and their description

Behaviour	Description
**Burrow**	Digging motions directed at the bedding, may include positioning a part or all of the body underneath bedding
**Climb**	The rat may have fore paws and rear paw(s) on the wall, usually occurring before or after a jump.
**Dart**	A sudden evasive movement from a still or slow walking position to a quick run or horizontal jump
**Eating**	The rat consumes the sucrose pellet. Usually, the forepaws are used to hold the pellet (positive trials only).
**Escape**	Leaping off the arena floor, attempting to pass over the arena wall. All four paws leave the ground.
**Explore**	Exploration of the environment, sniffing while moving the vibrissae and/or the head (head bobbing), while walking or stationary.
**Freeze**	Sudden suppression of movement in response to external stimuli. Body is rigid and alert, and not resting on the ground. The eyes are open and the ears might be perked.
**Groom**	Cleaning the fur and tail by mouth and/or paws including scratching
**Interact with object**	The rat touches, grabs, manipulates, or chews on the objects in the arena (post-it notes, cables, bedding)
**Non-intake**	Yawning or stretching.
**Other**	Any other behaviour
**Rear**	Maintenance of an erect posture without the forepaws touching the wall, usually associated with sniffing or head bobbing. The two forepaws leave the ground.
**Rest**	The rat is relaxed; the body is fully on the ground. The head is also on the ground. The eyes are closed or partially closed. The ears may be in a relaxed backward position.
**Stationary**	The rat is awake with eyes fully open and non-active (without sniffing or head bobbing) while maintaining a fixed posture. The rat is still reactive to stimuli through slight movements but does not rest full body weight on the floor.
**Walk**	Walking around without sniffing or interacting with objects in the arena.
**Wall grab**	Rearing with one or both forepaws placed on the wall but without obvious climbing, usually associated with sniffing or head bobbing.
