# Supplementary material for: Non-invasive assessment of positive affective state using infra-red thermography in rats
Source: Anim Welf. 2023 Sep 29;32:e66. doi: 10.1017/awf.2023.87 (PMC10951672; doi:10.1017/awf.2023.87)
Supplement: Wongsaengchan et al. supplementary material 5 — Wongsaengchan et al. supplementary material [file S0962728623000878sup005.pdf]

Table 2. GLMM analysis of the tail temperature difference from baseline using log likelihood tests. The table shows all the variables included in the models

Variables	All rats					Male rats					Female rats				
	2ΔLL	df	*p*	Estimate	SE	2ΔLL	df	*p*	Estimate	SE	2ΔLL	df	*p*	Estimate	SE
**Intercept**				-9.56E-01	5.09E-01				-1.30E+00	5.72E-01				7.67E-01	7.51E-01
**time**				3.66E-04	9.10E-04				-6.42E-04	1.06E-03				-4.02E-03	1.30E-03
**$I(time^2)$**				7.00E-07	5.00E-07				1.50E-06	6.00E-07				1.80E-06	7.00E-07
**Treatment**															
>Cheerios1				-2.35E-01	5.92E-01				1.52E-01	7.51E-01				-6.38E-01	9.33E-01
>Cheerios3				-1.23E-01	6.32E-01				4.34E-01	7.97E-01				-8.59E-01	1.00E+00
**Sex**															
>SexFemale				1.26E+00	4.81E-01										
**Body mass**						1.05	1	0.3054			1.16	1	0.2812		
**Location**	0.68	1	0.4082			0.04	1	0.8449			1.15	1	0.2840		
Location:Corner															
**Air Temperature**	0.06	1	0.8040			0.56	1	0.4540			0.76	1	0.3846		
**Humidity**	0.79	1	0.3753			1.93	1	0.1644			0.01	1	0.9627		
**TimeOfDay**	0.05	1	0.8149			0.11	1	0.7407			0.11	1	0.7378		
**Posture**	11.37	4	***0.0444***			8.06	4	0.1531			18.05	4	***0.0012***		
>Grooming				-1.83E-01	8.33E-02									-1.43E-01	1.20E-01
>Inactive				-1.99E-01	7.51E-02									-3.02E-01	1.07E-01
>Walking				-1.63E-01	7.33E-02									-1.74E-01	1.05E-01
>Jumping				-2.66E-01	9.30E-02									-2.74E-01	1.20E-01
**Treatment*time**	12.26	2	***0.0022***			25.55	2	***<.0001***			18.11	2	***0.0004***		
>Cheerios1:time				3.35E-03	1.13E-03				4.45E-03	1.50E-03				1.52E-03	1.68E-03
>Cheerios3:time				3.73E-03	1.21E-03				4.50E-03	1.61E-03				1.83E-03	1.80E-03
**Treatment*$I(time^2)$**	14.44	2	***0.0007***			20.23	2	***0.0002***			17.29	2	***0.0006***		
>Cheerios1:$I(time^2)$				-2.10E-06	6.00E-07				-3.00E-06	8.00E-07				-5.00E-07	9.00E-07
>Cheerios3:$I(time^2)$				-2.00E-06	6.00E-07				-2.80E-06	8.00E-07				-2.00E-07	1.00E-06
**Sex*time**	37.06	1	***<.0001***												
> time:Female				-5.82E-03	9.51E-04										
**Sex*$I(time^2)$**	22.58	1	***<.0001***												
>$I(time^2)$:Female				2.30E-06	5.00E-07										
**Sex*Treatment**	0.51	2	0.7747												
